# Acupuncture combined with methylcobalamin for the treatment of chemotherapy-induced peripheral neuropathy in patients with multiple myeloma

**DOI:** 10.1186/s12885-016-3037-z

**Published:** 2017-01-09

**Authors:** Xiaoyan Han, Lijuan Wang, Hongfei Shi, Gaofeng Zheng, Jingsong He, Wenjun Wu, Jimin Shi, Guoqing Wei, Weiyan Zheng, Jie Sun, He Huang, Zhen Cai

**Affiliations:** 1 Multiple Myeloma Center, Bone Marrow Transplantation Center, Department of Hematology, The First Affiliated Hospital, School of Medicine, Zhejiang University, No. 79 Qingchun Road, Hangzhou, 310003 China; 2 Present Address: Department of Hematology, Hematology Laboratory, Linyi People’s Hospital, Shandong University, Linyi, 276002 China

**Keywords:** Acupuncture, CIPN, Methylcobalamin, Multiple myeloma

## Abstract

**Background:**

Chemotherapy-induced peripheral neuropathy (CIPN) seriously affects the quality of life of patients with multiple myeloma (MM) as well as the response rate to chemotherapy. Acupuncture has a potential role in the treatment of CIPN, but at present there have been no randomized clinical research studies to analyze the effectiveness of acupuncture for the treatment of CIPN, particularly in MM patients.

**Methods:**

The MM patients (104 individuals) who met the inclusion criteria were randomly assigned into a solely methylcobalamin therapy group (500 μg intramuscular methylcobalamin injections every other day for 20 days; ten injections) followed by 2 months of 500 μg oral methylcobalamin administration, three times per day) and an acupuncture combined with methylcobalamin (Met + Acu) group (methylcobalamin used the same way as above accompanied by three cycles of acupuncture). Of the patients, 98 out of 104 completed the treatment and follow-ups. There were 49 patients in each group. The evaluating parameters included the visual analogue scale (VAS) pain score, Functional Assessment of Cancer Therapy/Gynecologic Oncology Group-Neurotoxicity (Fact/GOG-Ntx) questionnaire scores, and electromyographic (EMG) nerve conduction velocity (NCV) determinations. We evaluated the changes of the parameters in each group before and after the therapies and made a comparison between the two groups.

**Results:**

After 84 days (three cycles) of therapy, the pain was significantly alleviated in both groups, with a significantly higher decrease in the acupuncture treated group (*P* < 0.01). The patients’ daily activity evaluated by Fact/GOG-Ntx questionnaires significantly improved in the Met + Acu group (*P* < 0.001). The NCV in the Met + Acu group improved significantly while amelioration in the control group was not observed.

**Conclusions:**

The present study suggests that acupuncture combined with methylcobalamin in the treatment of CIPN showed a better outcome than methylcobalamin administration alone.

**Trial registration:**

China Clinical Trials Register (registration no. ChiCTR-INR-16009079, registration date August 24, 2016).

## Background

Multiple myeloma (MM) is a common hematologic malignancy and the incidence rate increases every year worldwide. Proteasome inhibitors such as bortezomib are commonly used for the initial treatment, as well as consolidation and maintenance therapies [1, 2]. However, chemotherapy-induced peripheral neuropathy (CIPN) during MM treatments is a dose-limiting side effect and the incidence rate of bortezomib-related neuropathy has been reported to be 30–60% [3, 4]. Common peripheral neuropathy symptoms in the distal limbs are symmetric sensory dysfunctions, with a variety of sensory losses such as glove or sock-shaped distribution, possibly associated with paresthesia and excessive pain. Other symptoms are movement disorders such as muscle weakness, muscle atrophy, diminished or disappeared limb and tendon reflexes, inability to fasten buttons as well as walking difficulties. In addition, autonomic nervous system disorders such as orthostatic hypotension, arrhythmia, bradycardia and other symptoms may occur.

Peripheral neuropathy is a key factor for drug dose and application duration restrictions, because patients often cannot tolerate symptoms, leading to a reduced drug dose and number of therapy cycles or even discontinuation of therapy. Therefore, reducing CIPN in MM treatments is a critical point for improving a patient’s quality of life and treatment outcome.

The therapy choices for CIPN treatments in MM patients are very limited but include neurotrophic drug treatment with methylcobalamin administered orally or as an intramuscular injection. The methylation of a functional group in methylcobalamin, a coenzyme of vitamin b12, enables drug availability and thereby promotes the metabolism of nucleic acids, proteins and lipids in nerve tissues. In addition, methylcobalamin stimulates cell lecithin synthesis, repairs damaged myelin and thereby improves nerve conduction velocity. First line treatments of neuropathic pain includes gabapentin, 5% lidocaine patches and opioid analgesics such as tramadol hydrochloride. Second line drugs include lamotrigine, carbamazepine and amitriptyline, as well as other antidepressants [5]. These drugs have various side effects, such as sedation, ataxia, dizziness, double vision, nausea and indigestion. The commonly used analgesics against neuropathic pain may work, but viable treatment options often do not completely relieve the symptoms.

However, when grades III-IV neurotoxicity occurs, the neurological symptoms will be partially relieved once the chemotherapy drug doses or therapy cycles are reduced, but inevitably the therapeutic effect on MM is also diminished.

Acupuncture, first mentioned in the 5th century BC, is part of traditional Chinese medicine and its effects, especially in pain control, have been confirmed in clinical trials, which led to the usage of acupuncture also in many other countries. A questionnaire of 180 patients with peripheral neuropathy showed that 30% of them choose acupuncture as an alternative method of pain control [6].

Studies on humans and animals have identified the neurochemical basis of acupuncture effects on brain functions. Acupuncture can stimulate receptors or cause the regular discharge of nerve fibers, leading to peripheral and central nervous system activation, resulting in the release of a variety of neurotransmitters [7]. The specific effect of acupuncture depends on the acupuncture point choice, the form of stimulation and the duration of the therapy [8]. Chinese acupuncture, an adjunct therapy, has gained increased attention in the medical field at home and abroad in recent years. Prospective clinical trials have demonstrated that acupuncture was effective in treating pain caused by diabetes as well as HIV virus infections [9–17], and various clinical trials have shown the effect of acupuncture in alleviating neuropathic pain in cancer patients [18, 19]. In addition, a case series has proven the efficacy of body acupuncture in treating patients with CIPN [20], and a pilot study demonstrated that acupuncture improved nerve conduction in peripheral neuropathy [21]. In recent studies, statistically and clinically significant reductions in subjective measurements of bortezomib-induced peripheral neuropathy (BIPN) were observed after acupuncture treatment [22, 23]. However, to date, there have been no randomized controlled clinical research to analyze the effectiveness of acupuncture in treating CIPN of MM patients.

Since previous research showed that acupuncture had good treatment effects on peripheral neuropathy of diabetes and HIV/AIDS patients, we hypothesized that acupuncture treatment of MM CIPN will also have positive therapeutic effects.

## Methods

### Patients

**Fig. 1:**
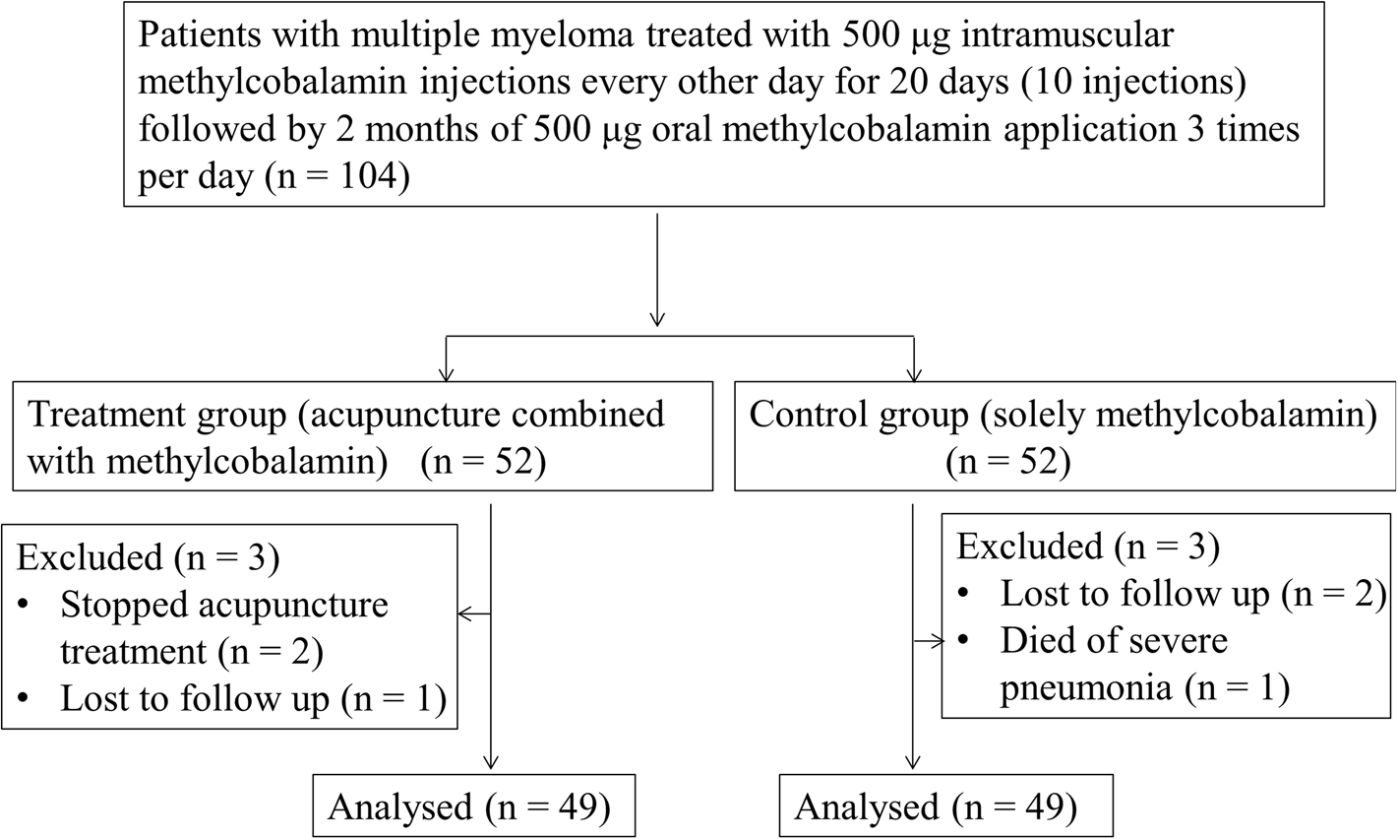
Flow chart of the present study

Four hundred twelve patients diagnosed with MM (not limited to the type or stage) were hospitalized for chemotherapy in our center between May 2010 and May 2014. The inclusion criteria were: diagnosed MM; baseline without peripheral neuropathy and peripheral neuropathy appeared after chemotherapy at grade II or above (according to the NCI CTCAE version 3.0 neuropathy severity assessment) [24]; EMG examinations showing disturbances in median and peroneal nerve conduction; platelet count greater than 30 × 10^9^/L; no history of methylcobalamin allergy; having discontinued chemotherapy within 3 months and were willing to accept new therapy and sign an informed consent form. The exclusion criteria were: pregnancy; severe heart, liver or kidney dysfunction or other severe diseases (e.g. malignancies); neuropathy caused by tumor compression, nutritional disorders or infections or causes other than chemotherapy; refusal to sign the informed consent form. The remaining 104 MM patients who met the inclusion criteria in our center were randomly divided into two groups. 98 out of 104 completed the treatment and follow-up. In the Met + Acu group, two patients stopped acupuncture treatment because of scheduled stem cell transplantation and one patient was lost to follow up. In the control group, two patients were lost to follow up and one patient died of severe pneumonia. Finally, 49 patients who were treated with acupuncture combined with methylcobalamin (Met + Acu group) and 49 patients only treated with methylcobalamin (control group) were included for the outcome analysis (Fig. 1).

### Treatments

**Fig. 2:**
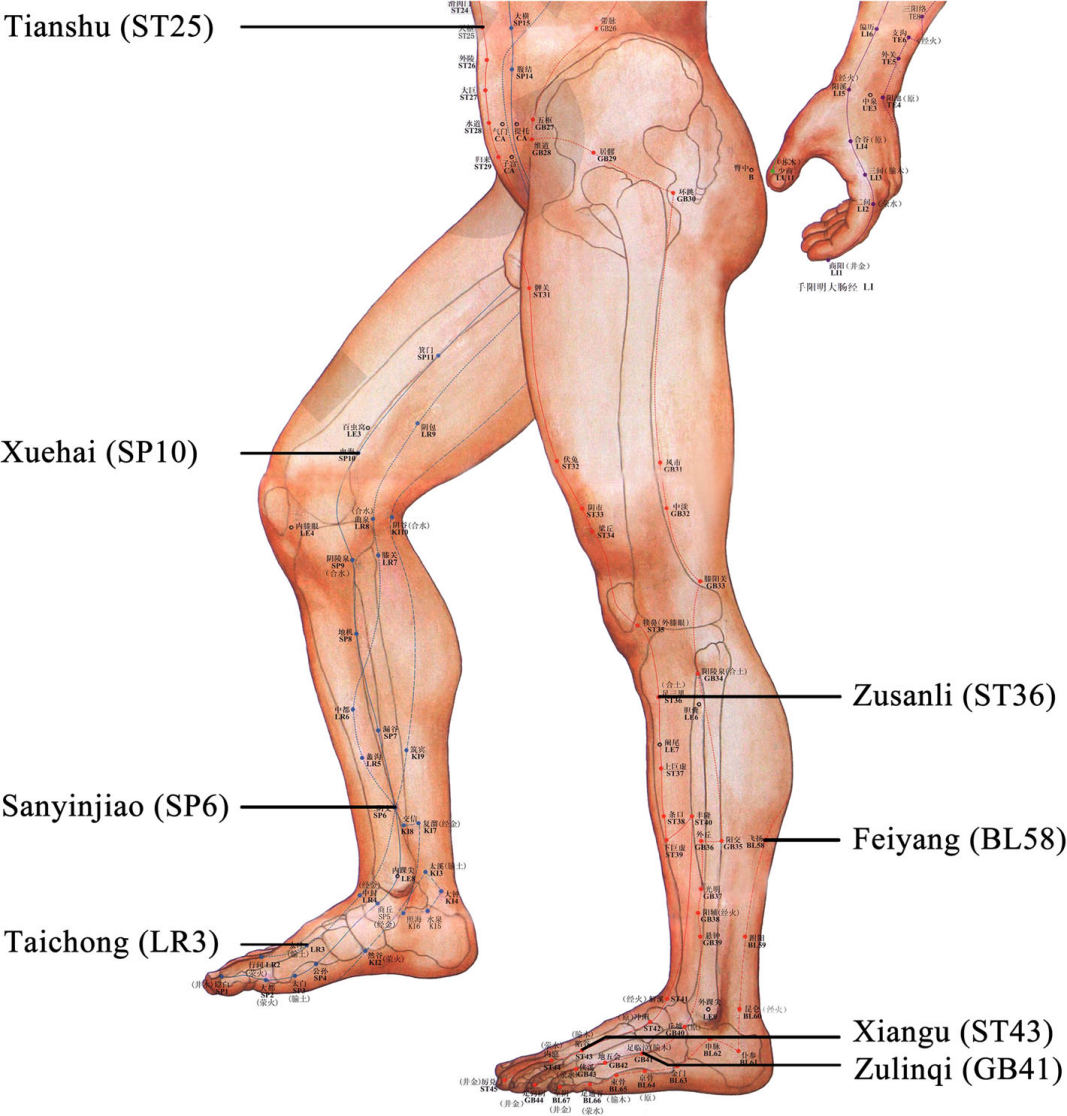
Scheme of acupuncture points on the legs, feet and ventral upper body

**Fig. 3:**
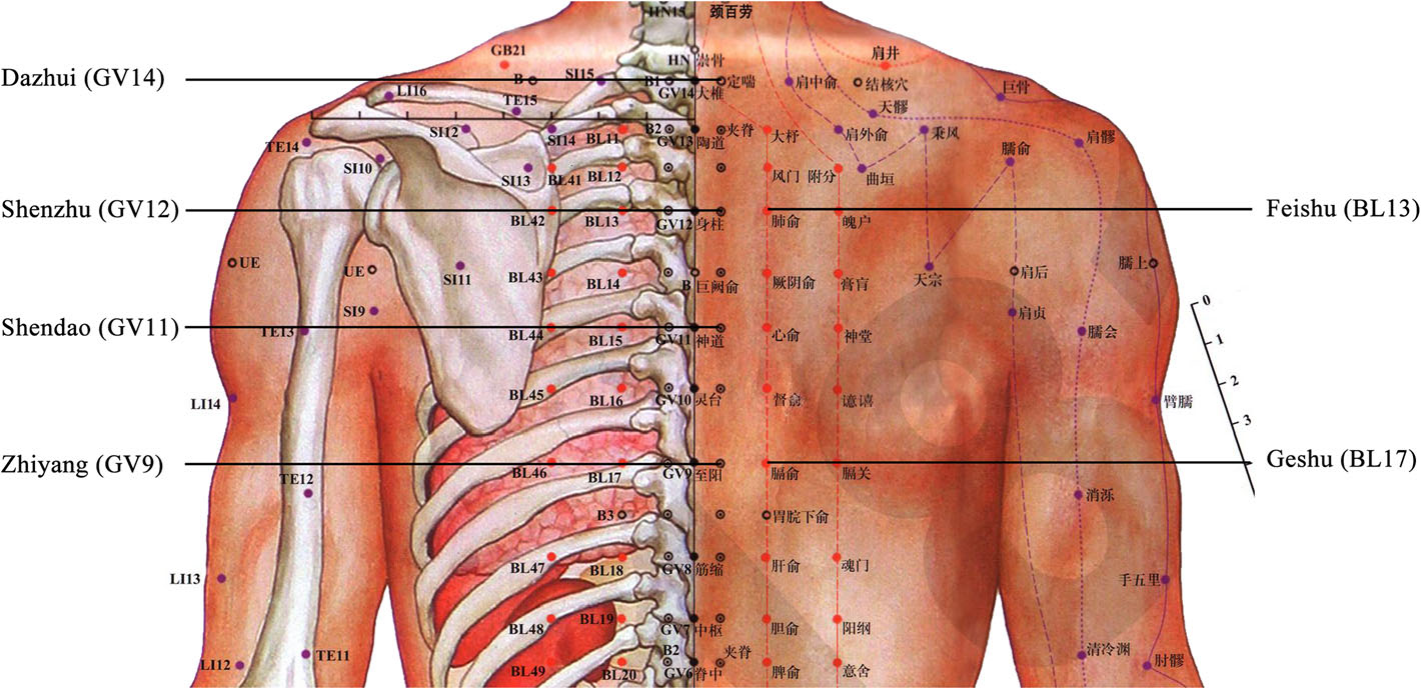
Scheme of acupuncture points on the dorsal upper body

The control group received only 500 μg methylcobalamin intramuscularly every other day, 10 times and thereafter 500 μg orally three times a day. The Met + Acu group received the same methylcobalamin application with an additional acupuncture protocol according to the neurohumoral mechanism theory of acupuncture [25]. In all cases, the acupuncture was performed by the same senior physician who had acupuncture experience for 15 years. Every patient received needles bilaterally in the following acupoints: Supine position: bilateral Taichong (LR3), Xiangu (ST43), Zulinqi (GB41), Sanyinjiao (SP6), Zusanli (ST36), Xuehai (SP10), Tianshu (ST25); Prone position: Dazhui (GV14), Shenzhu (GV12), Shendao (GV11), Zhiyang (GV9), Feishu (BL13), Geshu (BL17) and Feiyang (BL58) (Figs. 2 and 3). The first acupuncture was in prone position acupoints with needle retention, followed by supine position acupoints. An aseptic procedure was executed with disposable, stainless steel 30–32 gauge needles, which were implanted to a depth of 0.3–1.0 in. into the acupoints until the patient felt dull pain or de qi [26], and were left in place for 30 min. The acupunctures were done daily for 3 days, then once every alternate day for 10 days as a treatment cycle. Each cycle was repeated every 28 days and the complete treatment included three cycles.

### Evaluation standards

The validated Ntx extension of the Functional Assessment of Cancer Therapy/Gynaecologic Oncology Group/Neurotoxicity (FACT/GOG-Ntx) questionnaire [27] was used to investigate the patients’ daily activities and evaluate the degree of neuropathy. The questionnaire included 7 questions about physical well-being, 7 questions about social/family well-being, 6 questions about emotional well-being, 7 questions about functional well-being and 9 questions about additional concerns. The VAS pain score [28] was used to assess neuralgia. The bilateral NCV of the arms and legs was determined by the same professional technician before and after treatment using Nicolet Viasys Viking Select EMG NCS equipment from the USA. Skin surface electrodes were used to record the average of the motor conduction velocities (MCV) of the bilateral median and peroneal nerves as well as sensory nerve conduction velocities (SCV) of the bilateral median and the sural nerves. All evaluation measurements were carried out before and after treatments.

### CIPN severity assessment

CIPN was categorized according to the grading system published by Postma and Heimans (2000) [29].

### Statistical analysis

Statistical analyses were performed using GraphPad. Prism 5.01 statistical software. Assuming a mean value of the VAS score change was 2, standard deviation was 0.5 before and after treatment in the Met + Acu group, while the mean value of the VAS score change was 1.6; the standard deviation was 0 in the control group. A sample size of 41 in each group was considered to provide 95% power for detecting significant differences in the two groups (two-sided, α = 5%). To account for a 20% dropout, 104 patients in total (52 in each group) were included. The results are shown as the mean ± SEM. Between the two groups, single-factor analysis of variance (one-way ANOVA) and Tukey’s test were used for analyses while an independent sample t-test was used in one group. Statistical significance was considered at *P* < 0.05.

## Results

The characteristics of the treatment and control groups are shown in Table 1. There were no statistically significant differences between the 2 groups regarding the baseline characteristics.

**Table 1:** Baseline Characteristics of the MM patients

Characteristic	Met + Acu Group (*n* = 49)	Control Group (*n* = 49)
Sex		
Male	27	29^*^
Female	22	20^*^
Age (average)	62.46	65.29^*^
Type		
IgG	25	21^*^
IgA	13	14^*^
IgD	2	3^*^
Light chain (Kappa/lambda)	9	11^*^
PN Grade (CTCAE)		
Grade 2	20	19^*^
Grade 3	25	27^*^
Grade 4	4	3^*^
VAS scores	5.57 ± 0.26	5.50 ± 0.24^*^
FACT/the GOG-Ntx scores	36.48 ± 0.47	36.63 ± 0.55^*^

^*^ *P* > 0.05, compared between the Met + Acu and control groups before therapy

### VAS pain scores

After 3 cycles of therapy, the pain was significantly mitigated in the Met + Acu group, while the VAS pain scores decreased in 85.7% of the patients (42/49). Pain in the control group was also eased and the VAS pain scores decreased in 77.6% of these patients (38/49). However, the VAS pain scores in the Met + Acu group decreased more significantly compared to the control group (*P* < 0.01) (Table 2).

**Table 2:** VAS pain scores before and after treatment

Group	*n*	Before therapy	After therapy
Met + Acu Group	49	5.57 ± 0.257	3.23 ± 0.170^***^^△△^
Control Group	49	5.50 ± 0.244	4.25 ± 0.197^***^

^***^ *P* < 0.001, compared before and after therapy

^△△^ *P* < 0.01, compared between the Met + Acu and control groups

### Quality of life scores

Evaluated by FACT/the GOG-Ntx questionnaire scores, the nervous system symptoms improved significantly in the Met + Acu group (*P* < 0.001) after therapy, but not in the control group (*P* > 0.05), and the improvement was more significant in the Met + Acu group (*P* < 0.05) (Table 3).

**Table 3:** FACT/the GOG-Ntx questionnaire scores before and after treatment

Group	*n*	Before therapy	After therapy
Met + Acu Group	49	36.48 ± 0.470	32.98 ± 0.542^*** △^
Control Group	49	36.63 ± 0.551	35.17 ± 0.518

^***^ *P* < 0.001, compared before and after therapy

^△^ *P* < 0.05, compared between the Met + Acu and control groups

### Nerve conduction velocity

After treatments, there was no significant difference in MCV improvement in the Met + Acu group compared to the control group (*P* > 0.05). In contrast, before and after treatment in the Met + Acu group, the MCV of the bilateral median and peroneal nerves improved significantly after the acupuncture therapy (*P* < 0.05 and *P* < 0.01, respectively), while there was no obvious change in the control group (*P* > 0.05). The SCV of the sural nerve in the Met + Acu group improved significantly (*P* < 0.01), but there was no obvious change in the bilateral median nerve after the Met + Acu therapy (*P* > 0.05) (Table 4). Changes in the SCV of the sural and median nerve in the control group were not statistically significant (*P* > 0.05). Comparing the SCV after therapy between the two groups, the SCV of the sural nerve in the Met + Acu group was significantly superior to the control group (*P* < 0.01) (Table 4).

**Table 4:** Nerve conduction velocity before and after treatment

Group		*n*	MCV		SCV	
			Bilateral median nerve	Peroneal nerve	Bilateral median nerve	Sural nerve
Met + Acu group	Before treatment	49	49.81 ± 0.78	44.59 ± 0. 78	49.58 ± 0.73	43.99 ± 0.63
	After treatment	49	52.84 ± 0.81^*^	47.88 ± 0.67^**^	51.51 ± 0.64	46.87 ± 0.77^**△△^
Control group	Before treatment	49	50.43 ± 0.70	45.09 ± 0.71	49.98 ± 0.94	44.11 ± 0.60
	After treatment	49	51.05 ± 0.60	45.42 ± 0.76	50.40 ± 0.79	43.61 ± 0.51

^*^ *P* < 0.05, compared before and after therapy

^**^ *P* < 0.01, compared before and after therapy

^△△^ *P* < 0.01, compared between the Met + Acu and control groups

These data suggested that the treatment of Met + Acu improved the MCV and SCV (except for the median nerve SCV) in the Met + Acu group, while a solely methylcobalamin treatment in the control group had no effect on SCVs or MCVs.

## Discussion

To the best of our knowledge, this is the first randomized, controlled, prospective study on the use of acupuncture in the treatment of multiple myeloma patients with CIPN grades II–IV [29]. After 84 days (three cycles) of therapy, although methylcobalamin treatment alone was helpful in relieving pain and improving the quality of life, the study showed that acupuncture combined with methylcobalamin for the treatment of CIPN was significantly superior in providing pain relief (VAS pain scores) and life quality improvement (FACT/GOG-Ntx questionnaire scores). Our results are in agreement with previous reports that acupuncture has a beneficial effect on peripheral neuropathy and are consistent with the study of Schroder et al [21]; nerve conduction in the sural nerve was improved best in our study [20, 21]. The SCV of the median nerve did not change after a Met + Acu therapy, which might reflect the choice of acupuncture points, indicating that they have a major impact on the therapeutic effects [30].

According to traditional Chinese medicine (TCM) theory, the symptoms of CIPN are caused by the body’s failure to direct Qi (vital energy) and blood to the four limbs, resulting in sensory symptoms and impaired limb function while acupuncture restores body Qi and blood, and directs their flow to the extremities [20], which is supported by a studies which demonstrated that acupuncture led to vasodilation and enhanced blood perfusion [31, 32].

It has been suggested, that bortezomib mainly affects the dorsal root ganglia (DRG) of the primary sensory neurons leading to disturbed transcription, nuclear processing and transport, as well as cytoplasmic translation of mRNAs and histopathological changes in the DRG neurons. In addition, neural survival is compromised due to inhibition of nerve growth factor (NGF) transcription [33] and a highly significant correlation between the decrease in circulating levels of NGF and the severity of CIPN has been reported (*P* < 0.001) [34].

Previous animal studies noted that both protein and mRNA levels of glial cell line-derived neurotrophic factor (GDNF) and GDNF family receptor alpha-1 (GFRalpha-1) were upregulated in the DRGs after acupuncture [35].

However, another recent study found that acupuncture significantly changed the expression of 17 hypothalamic proteins in a rat neuropathic pain model [36]. Taken together, though enhanced blood perfusion as result of acupuncture has been proven in humans, other mechanisms of specific gene expression changes have so far only been investigated in animal models. It is also unclear whether acupuncture leads to histological changes, which might be evaluated in future studies with biopsy-analyses [21]. In addition, since the acupoints were established in TCM several centuries ago, analysis of acupoints with advanced techniques like MRI may lead to improved results.

There were no obvious unexpected side effects during the treatments of both groups, and puncture site infections or bleeding did not occur during the acupuncture process, suggesting that acupuncture is a safe treatment for CIPN in MM patients.

## Conclusions

In conclusion, our study revealed, in agreement with previous pilot studies, that acupuncture for the treatment of CIPN as adjunct therapy leads to a significantly improved outcome in MM patients.

## Abbreviations

CIPN: Chemotherapy-induced peripheral neuropathy
EMG: Electromyography
MCV: Motor conduction velocity
Met + Acu: Methylcobalamin + acupuncture
MM: Multiple myeloma
NCV: Nerve conduction velocity
SCV: Sensory conduction velocity
VAS: Visual analogue scale
